# Magnetic Resonance Imaging and Cerebrospinal Fluid Biomarker Clustering Defines Biological Subtypes of Alzheimer’s Disease

**DOI:** 10.3390/biomedicines13112632

**Published:** 2025-10-27

**Authors:** Rafail C. Christodoulou, Georgios Vamvouras, Maria Daniela Sarquis, Vasileia Petrou, Platon S. Papageorgiou, Ludwing Rivera, Celimar Morales, Gipsany Rivera, Evros Vassiliou, Elena E. Solomou, Sokratis G. Papageorgiou

**Affiliations:** 1 Department of Radiology, Stanford University School of Medicine, Stanford, CA 94305, USA; 2 Department of Mechanical Engineering, National Technical University of Athens, 15772 Zografou, Greece; gvamvouras@mail.ntua.gr; 3 Department of Medicine, Universidad de Carabobo, Valencia 2001, Venezuela; mdsarquis58@gmail.com; 4 Department of Medicine, University of Ioannina, 45110 Ioannina, Greece; md07010@uoi.gr; 5 2nd Department of Orthopaedic Surgery and Traumatology, Aghia Sophia Pediatric General Hospital, Thivon 3 Street, 15772 Athens, Greece; pplaton24@gmail.com; 6 Department of Medicine, American University of Antigua, Jabberwock Road, Osbourn 999152, Antigua and Barbuda; Ludwingr@auamed.net (L.R.); Celimarm@auamed.net (C.M.); Gipsanyr@auamed.net (G.R.); 7 Department of Biological Sciences, Kean University, Union, NJ 07083, USA; 8 Internal Medicine-Hematology, University of Patras Medical School, 26500 Rion, Greece; elenasolomou@hotmail.com; 9 1st Department of Neurology, Medical School, National and Kapodistrian University of Athens, Eginition Hospital, 15772 Athens, Greece; sokpapa@med.uoa.gr

**Keywords:** Alzheimer’s disease, MRI, CSF, clustering biomarkers

## Abstract

**Background/Objectives**: Alzheimer’s disease (AD) exhibits clinical and biological variability. This study aimed to identify MRI-defined subtypes reflecting distinct biological pathways of neurodegeneration and cognitive decline. **Methods**: We applied principal component analysis followed by k-means clustering (k = 3) on volumetric MRI data from 924 participants and validated clusters using cerebrospinal fluid (CSF) biomarkers (Aβ_42_, total tau, p-tau, CTRED, MAPres, glucose, CTWHITE). **Results**: Three major phenotypes emerged: (1) a tau/vascular limbic subtype with pronounced hippocampal and amygdala atrophy and elevated tau and CTRED levels; (2) a volume-preserved, low-amyloid subtype consistent with early-stage or cognitively resilient AD; and (3) a diffuse-atrophy subtype with high amyloid and tau burden and ventriculomegaly. Comparative analysis revealed progressive biological shifts from amyloid accumulation to tau aggregation and vascular compromise across these clusters. **Conclusions**: MRI-based clustering validated by CSF biomarkers delineates biologically meaningful AD endophenotypes. The results suggest a gradual cognitive decline driven by amyloid–tau–vascular interactions, supporting multimodal phenotyping as a practical approach for precision staging and intervention.

## 1. Introduction

Alzheimer’s disease (AD) is the most common neurodegenerative disorder, affecting 10% of the elderly population. It involves a gradual decline in cognition caused by neuropathological factors like amyloid-β accumulation and tau pathology aggregation [1]. Despite these standard mechanisms, AD is clinically and biologically heterogeneous, with patients experiencing a variety in the trajectory of cognitive impairment, atrophy patterns, and biomarker changes. AD heterogeneity poses significant challenges to diagnosis, prognosis, and therapeutic management, emphasizing the need to identify biologically distinct subtypes [2].

Structural MRI reveals reliable atrophy patterns in AD, including limbic-predominant, posterior cortical, and diffuse subtypes, differing in demographics, cognitive impairment, and prognosis [3]. These atrophy types produce distinct symptoms: temporal lobe atrophy impacts memory; subcortical atrophy (striatum, thalamus, cerebellum) links to memory decline and executive dysfunction; cortical atrophy affects memory only [4]. Behavioral and neuropsychiatric issues, from Mild Behavioral Impairment (MBI) to severe agitation, aggression, and psychosis, are associated with cognitive decline and linked to CSF biomarkers (ptau181, NFL, Aβ40/Aβ42) and neuroimaging [5,6]. Recent studies connect MBI with brain connectivity patterns and CSF tau and amyloid markers, supporting AD as a multidimensional syndrome [6,7]. PET imaging shows various AD features: FDG-PET hypometabolism in temporoparietal regions with moderate decline, limbic atrophy, and tau uptake, especially in the entorhinal cortex, which indicates tau pathology. Tau extends beyond the medial temporal lobe, with patterns like posterior, limbic, medial temporal-sparing, and left temporal [8]. Cortical hypometabolism focuses on the frontal lobe, impairing executive functions [9]. Cerebrospinal fluid (CSF) proteomics gained attention in AD clustering by revealing molecularly distinct subtypes [10]. Recent proteomics studies have identified three pathophysiological subgroups characterized by hyperplasticity, innate immune system dysfunction, and blood–brain barrier (BBB) dysfunction. Independent genetic analysis on these subgroups showed an excess genetic risk for AD and an increased risk for clinical progression [11]. This demonstrates that biological heterogeneity may complement structural heterogeneity, leading to personalized therapies and a better understanding of disease biology.

Multimodal clustering combines MRI, PET, CSF biomarkers, and cognition, emerging as promising. Clustering methods like SuStain show five AD groups with different risks and progression [12]. PET and atrophy-based subtypes overlap but are not identical, indicating hidden drivers. PET clusters outperform atrophy clusters in biomarkers and cognitive decline prediction, emphasizing the need to validate MRI-based atrophy phenotypes against biological markers [8]. AI-driven clustering has improved AD subtyping by combining imaging, molecular, and clinical data into single models. Deep learning techniques and multimodal fusion networks now allow for identifying hidden disease pathways and predictive endophenotypes [12,13,14]. This study extends the literature by applying unsupervised clustering to MRI features and testing if subgroups differ in CSF biomarkers like Aβ42, tau, CSF erythrocytes, glucose, and MAPres. We aim to see if MRI-based clusters match distinct biological phenotypes, improving AD classification. Unlike previous studies focusing on cortical atrophy or PET, our analysis combines MRI and CSF biomarkers, including CTRED, to assess if MRI-defined subtypes align with biological differences.

## 2. Materials and Methods

### 2.1. Participants and Data Source

Data were obtained from the Alzheimer’s Disease Neuroimaging Initiative (ADNI) database (https://adni.loni.usc.edu/, accessed on 1 September 2025). The dataset included structural T1-weighted 3D MPRAGE MRI scans and corresponding cerebrospinal fluid (CSF) biomarker measurements. MRI data were acquired according to the standardized ADNI MRI Core protocols across multiple scanner vendors and field strengths (1.5 T and 3 T). Typical acquisition parameters were repetition time (TR) ≈ 2300 ms, echo time (TE) ≈ 3 ms, inversion time (TI) ≈ 900 ms, flip angle = 9°, field of view = 256 × 240 mm^2^, and voxel size ≈ 1.0–1.2 mm isotropic, with minor vendor-specific variations detailed in the ADNI MRI Core documentation. All procedures complied with institutional review board (IRB) guidelines, and informed consent was obtained from all participants through ADNI.

#### 2.1.1. Segmentation and Volumetric Extraction

Segmentation was performed using FreeSurfer (v7.4.1; https://surfer.nmr.mgh.harvard.edu/, accessed on 1 September 2025), which automatically computed volumetric measures of interest. These included bilateral hippocampal, amygdala, and lateral ventricular volumes, as well as whole brain and estimated intracranial volume (ETIV).

#### 2.1.2. Computational Environment

Each MRI scan required approximately 6–8 h to be fully processed by FreeSurfer. To optimize throughput, processing was carried out on a Google Cloud Platform (GCP) Virtual Machine (N2-standard architecture, Intel^®^ Xeon^®^ Cascade Lake, 8 vCPUs, 32 GB RAM). This configuration enabled parallel execution of up to eight scans simultaneously. A fully automated shell-script pipeline managed job scheduling, error handling, and logging [15]. This ensured that computing resources were continuously utilized: whenever a process was completed or failed, a new scan was automatically queued. The cloud-based pipeline proved highly scalable, allowing continuous unattended execution. By leveraging parallelized segmentation and automated error handling, we eliminated idle processor time and maximized throughput. This approach enabled processing of thousands of scans within a timeframe that would not have been feasible on standard local hardware.

#### 2.1.3. Pipeline Structure

- Unzip DICOM data from raw archives.
- Convert DICOM images to NIfTI format using dcm2niix (v1.0.20220720).
- Submit up to eight scans concurrently to FreeSurfer for cortical and subcortical segmentation.
- Monitor job status and log one of three outcomes: (a) successful segmentation, (b) error (due to corrupted scans, extreme motion artifacts, or incomplete brain coverage), or (c) omission (non-brain scan types such as Localizer, GradWarp, Field Mapping, or N3).
- Upon completion, automatically append normalized volumes and metadata to a central repository.

#### 2.1.4. Quality Control and Exclusions

ADNI scans flagged as unsuitable (GradWarp, Localizer, Field Mapping, N3, or scaled scans) were excluded before segmentation. Acquisition dates between MRI and corresponding clinical/CSF assessments rarely matched exactly; therefore, a temporal tolerance of up to three months was permitted to maximize data inclusion. Outlier removal was performed at the volumetric stage: values falling below the 3rd percentile or above the 97th percentile, when physiologically implausible, were excluded to ensure model stability and reduce the impact of segmentation errors.

#### 2.1.5. Feature Selection and Dimensionality Reduction

Volumetric variables derived from FreeSurfer segmentation (hippocampal, amygdala, ventricular, whole-brain, and cortical regional volumes) were first compiled into a single dataset and inspected for missing or implausible values. Before analysis, all variables were z-standardized (mean = 0, standard deviation = 1) using DATAtab’s internal preprocessing routine to ensure comparability across regions of different absolute size. To reduce dimensionality and identify latent covariance structures among the volumetric measures, principal component analysis (PCA) was performed using DATAtab (v2025) [16] (https://datatab.net/, accessed on 10 September 2025). The covariance matrix extraction method was applied with Varimax rotation. Components were retained according to the Kaiser criterion (eigenvalue > 1) and visual inspection of the scree plot, ensuring that the selected components explained at least 85% of the total variance. The resulting principal component scores were exported and used as input features for the clustering analysis.

#### 2.1.6. Clustering Procedure

Unsupervised clustering was performed using the K-Means algorithm module of DATAtab, which was applied to the retained principal component scores. The Euclidean distance metric and k-means++ initialization were utilized with 10 random starts and a maximum of 300 iterations per run to ensure convergence. The optimal number of clusters (k = 3) was determined via the elbow method, which balances explained variance (within-cluster inertia) against model simplicity. This included plotting the total within-cluster variance against the number of clusters, and the point where the rate of improvement sharply decreases, forming an “elbow” in the curve, indicates the optimal k beyond which additional clusters add little explanatory value. The stability of the three-cluster solution was confirmed by repeating the analysis under different random seeds, which yielded consistent results. Final cluster assignments were used to compute centroids and descriptive statistics of key volumetric features for biological interpretation.

#### 2.1.7. CSF Biomarker Analysis

CSF biomarkers included amyloid-β42 (Aβ42), total tau (Tau), phosphorylated tau (pTau), CTRED, CTWHITE, MAPres (defined in Equation (1)), and glucose. Raw values were visually inspected for outliers and distributional skewness. Since most variables deviated from normality, non-parametric testing was applied. To evaluate whether MRI-defined clusters corresponded to distinct biological profiles, a repeated-measures Kruskal–Wallis analysis was conducted within DATAtab. Main effects of biomarker, cluster, and their interaction were assessed. Bonferroni-corrected post hoc pairwise comparisons followed significant omnibus effects. Effect sizes (η^2^) were reported to quantify the proportion of variance explained. Biomarker results were summarized using cluster-wise arithmetic means and visualized through DATAtab’s integrated plotting interface.

(1) $$MAPres=\frac{2\cdot DBP+SBP}{3}$$

#### 2.1.8. Software and Statistical Environment

PCA and K-Means clustering were conducted using the DATAtab software. Statistical analyses for biomarker validation were performed using DATAtab’s hypothesis testing framework. All figures and tables were generated directly from these analyses.

## 3. Results

### 3.1. Principal Component Analysis

Principal component analysis (PCA) was applied to all volumetric MRI variables, including hippocampal, amygdala, ventricular, whole-brain, and major cortical regions (entorhinal, precuneus, inferior parietal, and middle temporal cortices). All variables were standardized prior to analysis, as mentioned in Section 2.1.5. The analysis extracted seven principal components, collectively explaining 86.55% of total variance (Table 1).

The first component (PC1) accounted for 41.65% of variance and was characterized by strong positive loadings for total brain volume (0.91), estimated intracranial volume (0.86), and medial-temporal structures such as the hippocampus and amygdala bilaterally (0.71–0.81) (Table 2). This component represents a global atrophy axis, reflecting overall brain size and limbic integrity.

The second component (PC2, 13.98%) primarily captured temporo-parietal cortical variance, driven by the middle temporal, inferior parietal, and precuneus cortices, suggesting a cortical-atrophy gradient independent of global volume. PC3 (9.46%) represented ventricular enlargement contrasted against parenchymal volume, whereas PC4–PC7 (7.39–3.01%) accounted for smaller regional variations across frontal and occipital structures.

**Figure 1:**
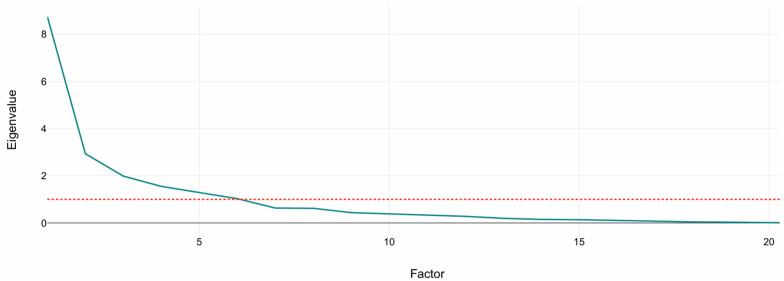
Scree plot of principal component eigenvalues. The plot shows the variance explained by each component from standardized volumetric MRI variables. The dashed line marks the Kaiser criterion (eigenvalue = 1). The sharp drop after the third component indicates that the first six to seven components capture most of the structural variance used for clustering.

Figure 1 shows the scree plot of eigenvalues. A sharp drop between PC1–PC3 followed by a gradual flattening supports the retention of the first six to seven components, which exceed the Kaiser threshold (eigenvalue = 1). These retained components were subsequently used as input features for the K-Means clustering analysis described in Section 3.2.

### 3.2. Clustering Outcomes

**Figure 2:**
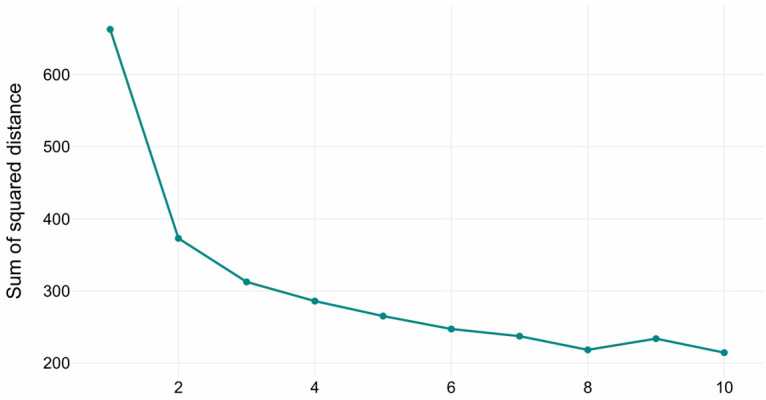
Elbow method diagram for K-Means.

The optimal number of clusters in the K-Means clustering was determined via the elbow method (see Figure 2). The inflection points in the curve indicated that the optimal number of clusters is three, representing the trade-off between explained variance (inertia reduction) and model parsimony (avoiding over-segmentation). The three clusters with sizes of 482, 292, and 150 samples, respectively. Cluster centroids reflected distinct volumetric profiles across hippocampal, amygdala, ventricular, and global brain measures.

Three distinct clusters were revealed, with *n* = 482, 292, and 150, respectively, totaling *n* = 924. The centroids highlighted clear volumetric differences. Cluster 1 was defined by intermediate atrophy, Cluster 2 by preserved volumes, and Cluster 3 by severe atrophy combined with ventricular enlargement. Specifically:

- **Cluster 1 (Limbic Predominant)**: Hippocampal volumes ~2430–2518 mm^3^, amygdala ~797–878 mm^3^, ventricles ~21,000–20,500 mm^3^, brain volume ~1,276,402 mm^3^.
- **Cluster 2 (Moderate Atrophy)**: Hippocampal ~3639–3722 mm^3^, amygdala ~1300.6 mm^3^, ventricles ~23,600–22,000 mm^3^, brain volume ~1,300,469 mm^3^.
- **Cluster 3 (Severe Atrophy with Enlarged Ventricles)**: Hippocampal ~3196–3229 mm^3^, amygdala ~1271–1508 mm^3^, ventricles markedly enlarged (~57,804–55,129 mm^3^), brain volume ~989,565 mm^3^.

### 3.3. CSF Biomarker Profiles

To evaluate whether the MRI-defined clusters differed in their cerebrospinal fluid (CSF) biomarker composition, seven biomarkers were analyzed: Aβ42, total tau (Tau), phosphorylated tau (pTau), CTRED, CTWHITE, MAPres, and glucose. A repeated-measures Kruskal–Wallis test revealed statistically significant main effects of biomarker (*p* < 0.001), cluster (*p* < 0.001), and a significant biomarker × cluster interaction, indicating that biomarker patterns varied systematically across the three clusters.

Post hoc pairwise comparisons with Bonferroni correction showed that Clusters 2 and 3 did not differ significantly (*p* = 1.00), whereas Cluster 1 differed from both Clusters 2 and 3 (*p* < 0.001 for both contrasts). This pattern suggests that the limbic-predominant group (Cluster 1) has a distinct biological profile compared to the other two.

Cluster-wise mean values are presented in Table 3. The Tau/CTRED-high profile in Cluster 1 is characterized by markedly elevated Tau, pTau, and CTRED levels, consistent with tauopathy and erythrocyte-related or vascular mechanisms. Cluster 2, which exhibited preserved brain volumes, showed higher Aβ42 and lower Tau/pTau, representing a low-amyloid, low-tau phenotype suggestive of earlier disease stages or slower progression. Cluster 3, in contrast, demonstrated reduced Aβ42 combined with elevated Tau/pTau, forming a tau-dominant, amyloid-low profile typical of more advanced pathology.

**Figure 3:**
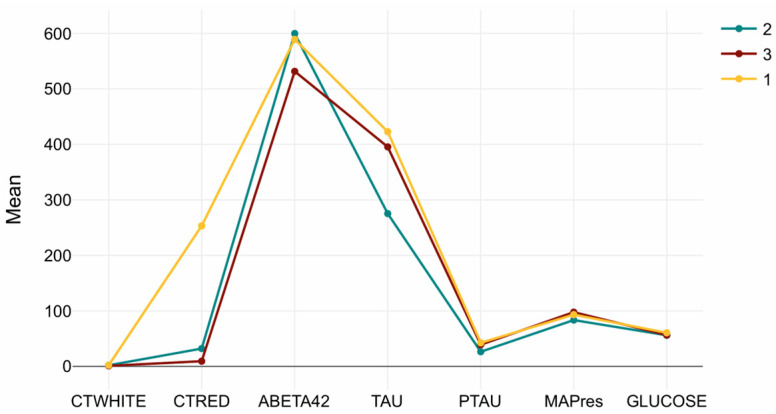
Cluster-wise CSF biomarker signatures. Mean (±SD) values of Aβ42, Tau, pTau, CTRED, CTWHITE, MAPres, and glucose across the three MRI-defined clusters. Cluster 1 (Tau/CTRED-high), Cluster 2 (Aβ42-high/Tau-low), and Cluster 3 (Tau-dominant, Aβ42-low) display distinct biochemical profiles, while MAPres, CTWHITE, and glucose remain relatively stable.

MAPres, CTWHITE, and glucose showed minimal variation across clusters, reflecting that vascular or metabolic markers were relatively stable compared to amyloid and tau indicators. Figure 3 summarizes the biomarker distributions for each cluster, illustrating the distinct molecular signatures underlying the MRI-based subtypes.

### 3.4. Subtype Interpretation

Integration of MRI volumetrics and CSF biomarkers delineated three biologically meaningful Alzheimer’s disease subtypes:

1. **Tau/CTRED-high (Cluster 1)**: Intermediate atrophy with elevated Tau/pTau and higher erythrocyte load.
2. **Aβ42-high/Tau-low (Cluster 2)**: Preserved volumes with higher Aβ42 and lower Tau/pTau, consistent with a comparatively lower amyloid and tau burden.
3. **Tau-high/Aβ42-low (Cluster 3)**: Marked ventricular enlargement with a tau-dominant CSF profile (elevated Tau/pTau) and reduced Aβ42, indicating greater pathological burden than Cluster 2.

Notably, although the overall post hoc test on the cluster factor found no global difference between Clusters 2 and 3, their biomarker patterns diverged as described.

## 4. Discussion

### 4.1. Overview of Cluster Characteristics and Biological Validation

Unsupervised clustering on volumetric MRI features was applied, and the subgroups’ biological relevance was assessed through CSF biomarkers. Three clusters were identified: (1) Tau/CTRED-high group with limbic atrophy, (2) a volume-preserved group with low amyloid burden (high Aβ42) indicative of early disease stages, and (3) a Tau-focused, high amyloid (low Aβ42) group showing severe diffuse atrophy and ventriculomegaly. CSF analysis confirmed higher Tau and CTRED levels in the limbic-atrophy cluster, while the volume-preserved group exhibited greater amyloid burden. These results indicate that MRI-based clusters correspond to meaningful biological profiles in AD.

CSF biomarker comparisons confirmed significant differences between the MRI-defined clusters. Biomarkers accounted for the most critical portion of variance, with clusters contributing a more negligible yet meaningful effect, and their interaction was also statistically significant. Post hoc analyses revealed that Cluster 1 exhibited notably higher levels of Tau (total, pTau) and CTRED compared to Clusters 2 and 3, which did not differ from each other. This pattern indicates that the limbic-atrophy cluster is biologically distinct, marked by tauopathy and vascular/erythrocyte mechanisms. In contrast, the preserved-volume cluster represents an amyloid-predominant, early-stage phenotype, whereas the diffuse atrophy cluster reflects a tau-dominant, advanced neurodegeneration characterized by ventriculomegaly.

**Figure 4:**
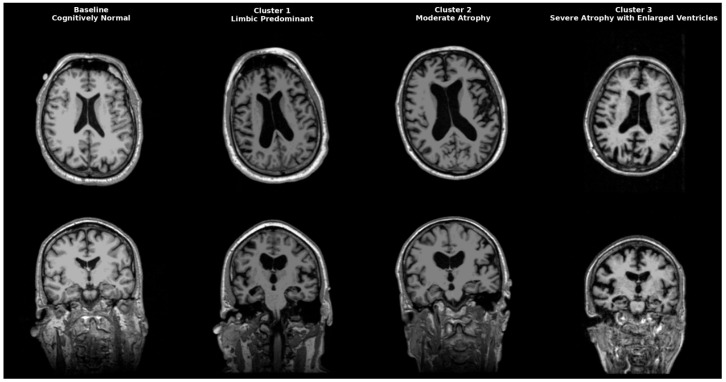
Representative MRI scans of Alzheimer’s disease subtypes identified by clustering. Axial (top row) and coronal (bottom row) T1-weighted MRI images illustrate a cognitively normal baseline (leftmost column) compared to three distinct AD clusters. Cluster 1 (Limbic-predominant) shows marked hippocampal and amygdala atrophy with relative cortical preservation. Cluster 2 (Volume-preserved, moderate atrophy) demonstrates largely preserved cortical and subcortical volumes despite abnormal CSF biomarkers. Cluster 3 (Diffuse atrophy with ventriculomegaly) exhibits widespread cortical thinning and enlarged ventricles (hydrocephalus ex vacuo), consistent with advanced disease.

AD is a heterogeneous neurodegenerative disorder that could be classified anatomically and biologically into distinct groups. Neuropathological studies post mortem first identified limbic predominant, hippocampal-sparing, and typical AD. Interestingly, patients with the hippocampal-sparing type died earlier and were men, while those with the limbic-predominant type were women who died older [17]. Neuropathological subgroups were later replicated in imaging cohort studies, solidifying AD variety in atrophy patterns. Representative MRI scans of each identified cluster are shown in Figure 4, highlighting the limbic-predominant (Cluster 1), volume-preserved (Cluster 2), and diffuse atrophy with ventriculomegaly (Cluster 3) subtypes, alongside a cognitively normal baseline for comparison. While our clustering primarily focused on structural and CSF-derived biomarkers, future research might include epigenetic and transcriptomic signatures to improve endophenotype definitions. Recent studies indicate that DNA methylation, microRNA regulation, and gene expression networks impact amyloid processing, tau phosphorylation, and neuroinflammation, influencing regional vulnerability in AD [18,19,20]. These molecular aspects could increase biological accuracy and help link imaging-based subtypes with their cellular mechanistic profiles.

### 4.2. Biological and Clinical Profiles of Identified Clusters

Cluster 1 in our analysis mirrors the limbic predominant AD subtype, characterized by severe hippocampal and amygdala atrophy alongside tau and CTRED elevation. Prior work shows that CSF tau markers negatively correlate with hippocampal volume tau (R = −0.53), ptau (R = −0.56), while no correlation was found with CSF Aβ42 levels, suggesting that tau markers may reflect neuronal loss better [21]. Moreover, BBB breakdown tends to be localized early and more severe in the hippocampus, providing a plausible route for red blood cells (RBC) to enter CSF in structures undergoing neurodegeneration [22]. Products resulting from RBC breakdown (Hb, Fe2+) disturb hippocampal metabolism, leading to iron imbalance. Notably, hemoglobin subunits correlated with increased tau deposition in the brain, and AD converters showed significantly higher baseline Hb levels [23]. Together, these data support our finding that the Tau/CTRED-high cluster reflects a tau and vascular/erythrocyte-associated limbic system degeneration phenotype [24]. Clinically, the Tau/CTRED high cluster presents with classic amnestic syndrome driven by hippocampal and amygdala degeneration and elevated tau levels. Neuropsychiatric symptoms such as depression, anxiety, apathy, and irritability are common and often result from amygdala atrophy because of its role in emotional perception and recognition [25]. Demographically, women are over-represented in limbic predominant AD and APOE ε4, related to greater structural atrophy and memory impairment within the cluster [17,26].

Cluster 2 (Low Amyloid Burden, Volume preserved) resembles a low amyloid volume-preserved subtype, where amyloid pathology is present, but volumes are maintained, and tau elevation is minimal, consistent with early-stage disease and slower progression. Previous literature defined this cluster as a no atrophy AD subtype, which is often found in younger, more often female individuals with abnormal CSF biomarkers but no structural atrophy despite memory deficits. It could indicate an early-stage disease or slow progressors [27]. Although this cluster may seem controversial, pathology with no atrophy may be explained by the cognitive reserve hypothesis, where individuals with greater education or cognitive engagement can maintain functional capacity longer despite underlying pathology [28]. However, prior studies have shown that patients with higher cognitive reserve often present with more pronounced atrophy at diagnosis, as symptoms emerge only once structural damage has advanced [29,30]. Another possible explanation may be that this subgroup’s low amyloid burden is not enough to cause atrophy, but affects the neurons’ functional abilities by impairing signaling pathways, ultimately leading to clinical symptoms [31]. In addition, Cluster 2 (Table 4) displayed a distinctive descriptive profile, with all participants affected by hypertension (100%), none with diabetes (0%), and a comparatively lower prevalence of APOE ε4 carriers (66.7%) than in Clusters 1 and 3. Although these differences were not subjected to statistical testing, they suggest a possibly distinctive vascular and genetic profile that could affect the slower disease progression seen in this group. This pattern resembles early or slow-progressing phenotypes, consistent with recent AI-based classification work demonstrating that biomarker-driven models (Aβ42, pTau, and vascular factors) can detect subtle disease states at the prodromal stage [32].

Lastly, cluster 3 (Tau Focused, High Amyloid, Diffuse Atrophy) aligns with typical AD, where widespread atrophy and ventricular enlargement are associated with advanced disease. Aβ42 is the main component of amyloid plaques, and current hypotheses suggest that lower CSF levels, as shown in this subgroup, suggest its aggregation in the brain [33].On top of that, a high amyloid burden enhances tau hyperphosphorylation, misfolding, and aggregation into neurofibrillary tangles, resulting in high tau levels, leading to neuronal death and cognitive decline [34]. Patients of this subgroup present clinically with multidomain cognitive impairment, neuropsychiatric symptoms like anxiety, depression, or psychosis, and progressive loss of daily functioning, which is a hallmark of AD [35].The accompanying ventricular enlargement results from diffuse brain atrophy (hydrocephalus ex vacuo). It is a radiological feature that requires careful distinction from normal pressure hydrocephalus (NPH), where ventriculomegaly arises from impaired CSF dynamics. Importantly, co-morbidity of NPH and AD is not rare, with epidemiological studies reporting overlap rates of approximately 25% [36]. AD patients primarily exhibit amnestic and cortical syndromes (impaired executive function, cognitive and behavioral changes). NPH is characterized in 50–75% of cases by the triad of cognitive impairment, urinary incontinence, and gait disturbances [37]. In the classical presentations, however, the two could present similarly in clinical practice. In general, NPH is marked by disproportionate ventricular enlargement on MRI. NPH radiological criteria help differentiate it from AD, but discussing these criteria is beyond this article’s scope [38]. Differentiating the two is crucial, as NPH may respond to shunting, unlike the progressive course of AD. Because of the common co-morbidity, it is essential not to overlook cases where NPH is also present.

Notably, integrating CSF CTRED into clustering frameworks is a novel contribution. Although most subtype studies focus on amyloid and tau, our results show that CTRED is explicitly elevated in the tau-dominant cluster. This indicates that the vascular or blood–brain barrier may contribute to heterogeneity, supporting the idea that vascular and inflammatory processes fueled by erythrocyte byproducts and toxins from BBB penetration affect neurodegeneration in AD [39,40]. Inclusion of metrics like CTRED or MAPres may, therefore, help capture distinct phenotypes that are not solely explained by amyloid-tau dynamics. Clinically, integrating these vascular markers into diagnostic procedures could enhance traditional amyloid and tau tests by pinpointing patients with a greater vascular–inflammatory component. This approach might enable earlier detection of mixed or atypical cases, assist in personalized management of vascular risk, and guide therapeutic choices such as restoring BBB integrity or reducing oxidative and erythrocyte-driven stress. In the end, vascular-based phenotyping could facilitate a more comprehensive, mechanistically informed strategy for diagnosis and treatment planning in AD.

### 4.3. From Imaging Phenotypes to Personalized Alzheimer’s Care

MRI-Biomarker clustering findings also contribute to broader discussions about the predictive value of imaging subtypes. Studies using tau PET indicate that tau spread often corresponds with, but does not fully match, MRI-based subtypes. Moreover, tau-defined clusters tend to predict clinical decline [41,42]. Our results show MRI-based clusters can reveal distinct phenotypes when validated against CSF biomarkers, supporting MRI as an accessible tool for precise phenotyping.

Beyond precise phenotyping, given the multifactorial and complex pathophysiology of AD, grouping patients by volumetric and biomarker characteristics opens avenues for personalized medicine [43]. Alzheimer’s is not a unitary disease but a syndrome displaying heterogeneity across molecular and clinical dimensions, establishing the need for tailored diagnostic and therapeutic strategies [44]. Studies have shown that combining imaging and biomarkers yields more precise phenotypes than single-marker approaches [45]. Artificial intelligence (AI) combined with multiple modalities of biomarkers can aid stratification and enrollment in clinical trials, reshaping the future of personalized medicine [46,47]. For instance, due to its strong connection with iron dyshomeostasis, the TAU/CTRED limbic predominant subtype might be eligible for iron chelation apart from tau-specific clinical trials [48,49]. Although enrollment in these trials must be considered only after shared decision making, since the deferiprone randomized clinical trial has been shown to worsen cognitive decline, even though it lowers hippocampal QSM [50]. Given the high metabolic vascular risk factors that tend to exist in the limbic predominance and subtypes, these should be addressed by lowering cholesterol, managing blood pressure, and increasing activity levels [24,28,51]. In addition, the slower progression of cluster 2 (low amyloid, volume preserved) suggests that it may benefit from lifestyle interventions and vascular risk optimization, consistent with multidomain prevention trials such as FINGER [52]. Symptomatic therapy, combined with regular monitoring of cognition and biomarkers, is an additional strategy and highlights that this group is an essential target for early intervention.

**Figure 5:**
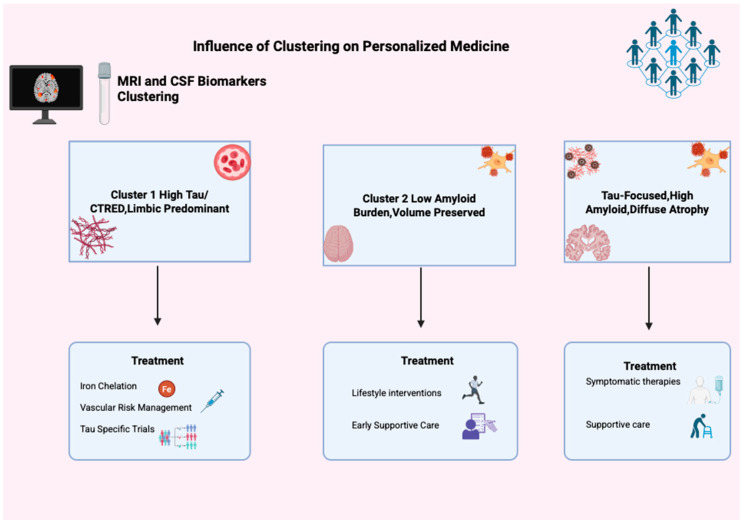
**Influence of clustering in personalized medicine.** This figure illustrates clustering’s impact on personalized Alzheimer’s medicine using MRI and CSF biomarkers, identifying three groups: Cluster 1 (High Tau/CTRED, Limbic Predominant), Cluster 2 (Low Amyloid, Volume Preserved), and Cluster 3 (Tau-Focused, High Amyloid, Diffuse Atrophy). Each has unique treatment options like iron chelation, tau trials, lifestyle changes, and support, showing clustering’s role in tailored AD treatment.

Finally, managing the typical AD cluster involves optimizing vascular and metabolic health, starting or maintaining symptomatic treatments such as cholinesterase inhibitors and memantine, and managing neuropsychiatric symptoms through both medications and behavioral approaches. Supportive care, including physical and occupational therapy, fall prevention, and caregiver support, is essential as patients’ independence declines [53,54]. This underscores the need to customize care (Figure 5) plans based on biomarker profiles, disease stage, and functional ability.

### 4.4. Limitations of the Study

This study comes with some limitations. First, the cross-sectional design restricts conclusions about longitudinal progression or conversion risk. PCA and K-Means clustering rely on simplifying assumptions that may only partly hold for biological data. K-Means assumes spherical clusters with similar variance and Euclidean separability, while PCA presumes linear feature relationships, although neurodegenerative processes are often nonlinear. The retained components explained 86.55% of the variance, yet the remaining variance may still contain biologically relevant information. Cluster stability was confirmed by repeating K-Means with multiple random initializations, but broader bootstrapped validation would further support robustness. Moreover, discretizing Alzheimer’s disease into distinct clusters may oversimplify its continuous spectrum and overlook intermediate phenotypes. CSF biomarkers represent global averages and may not capture regional pathological variability. Future studies combining nonlinear or density-based methods with region-specific biomarkers could refine these subtypes and strengthen biological interpretability.

Lastly, in our sample, the sex distribution across clusters did not fully match previous reports. For instance, the limbic-predominant and no-atrophy subtypes are typically described as more prevalent in women, yet our group showed fewer females in these categories. This difference might be due to sample size and composition rather than actual biological variation, but it underscores the importance of confirming subtype demographics in larger samples. While statistically significant, the cluster effect size (η^2^ = 0.01) indicates only a small amount of variance explained, highlighting the need for further validation with larger, independent datasets and cohorts. While we included Aβ42, Tau, pTau, CTRED, MAPres, glucose, and CTWHITE, incorporating multimodal data such as PET imaging, proteomics, transcriptomics, and cognitive outcomes could enhance subtype classification. It is important to note that CTRED is a relatively new biomarker without standardized thresholds or extensive validation in AD. Therefore, its interpretability should be considered preliminary until broader replication is available.

### 4.5. Future Directions

Future research should conduct longitudinal studies to observe how MRI–CSF–defined subtypes change over time and to clarify their prognostic and biological trajectories. Tracking alterations in structural and biochemical markers will help determine if patients transition between tau-vascular, amyloid-predominant, or diffuse-atrophy phenotypes as the disease progresses. A detailed assessment of neuropsychiatric symptoms within each cluster is also essential, especially since these symptoms often manifest as MBI that predicts cognitive decline and correlates with biomarkers of AD, such as plasma phosphorylated tau, neurofilament light chain (NfL), and amyloid profiles, underscoring their biological significance [7]. Additionally, integrating multi-omic data (epigenetics, transcriptomics, proteomics) will enhance endophenotype refinement by linking imaging clusters to molecular information. Lastly, since CTRED is still new, further research should establish thresholds, reliability, and sensitivity to treatment and vascular changes. Overall, these steps will deepen our understanding of MRI–biomarker phenotyping and support the development of personalized, multidimensional models in AD.

## 5. Conclusions

This study shows that unsupervised clustering of MRI features, validated with CSF biomarkers, can identify distinct AD phenotypes. These biomarker signatures highlight AD heterogeneity and support including vascular and erythrocyte markers like CTRED in refining subtypes. Recognizing these biologically distinct subtypes may enable precision medicine, allowing early identification of at-risk individuals and targeted interventions tailored to each disease mechanism. However, the cross-sectional design limits understanding of progression, requiring replication in larger, diverse cohorts. Future multimodal studies must validate and refine these clusters, aiding clinical trial stratification and personalized treatment. Overall, AD comprises multiple biological and structural endophenotypes, and combining MRI with CSF biomarkers transforms AD management, paving the way for more precise disease characterization and care.

## Figures and Tables

**Table 1:** Explained variance percentage per PCA component.

Component	1	2	3	4	5	6	7
**Explained Variance [%]**	41.65	13.98	9.46	7.39	6.15	4.91	3.01

**Table 2:** Dominant loadings for the first principal component.

Volumetric Variables	Loading Value
Total brain volume	0.91
ETIV	0.86
RH Middle Temporal	0.83
RH Hippocampus	0.81
RH Amygdala	0.78
LH Amygdala	0.75
RH Inferior Parietal	0.75
LH Hippocampus	0.71
LH Entorhinal	0.71
LH Middle Temporal	0.68
RH Precuneus	0.68
LH Inferior Parietal	0.61

**Table 3:** Arithmetic means per cluster.

	CTWHITE	CTRED	ABETA42	TAU	PTAU	MAPres	GLUCOSE
**2**	2.01	32.11	599.81	275.34	26.4	83.47	56.09
**3**	1	9.34	531.62	395.55	39.13	97.72	56.47
**1**	2	253.24	589.21	422.88	42.51	93.27	60.55
**Overall**	1.5	90.38	562.21	382.26	37.87	93.72	57.69

**Table 4:** Clusters Characteristics Description.

Variable	Cluster 1	Cluster 2	Cluster 3	Overall
**Subjects (%)**	38.5	11.5	50.0	100
**Age, years mean and SD**	71.3 (9.6)	77.0 (4.4)	75.1 (6.1)	73.8 (7.6)
**Females (%)**	40.0	0.0	84.6	57.7
**Education, years mean and SD**	16.4 (2.3)	16.0 (4.0)	13.8 (2.2)	15.1 (2.7)
**Hypertension (%)**	70.0	100.0	69.2	73.1
**Diabetes (%)**	40.0	0.0	38.5	34.6
**APOE4 carriers ≥ 1 (%)**	80.0	66.7	84.6	80.8
**Aβ42, pg/mL mean**	589.21	599.81	531.62	578.6 (217.0)
**Tau, pg/mL mean**	422.88	275.34	395.55	375.0
**pTau, pg/mL mean**	40.7	26.4	39.13	37.8
**MMSE mean and SD**	23.6 (1.8)	23.0 (1.7)	23.2 (3.6)	23.3 (2.8)
**CDR-SB mean and SD**	4.3 (1.5)	2.7 (0.6)	4.3 (1.3)	4.1 (1.4)

## Data Availability

The data presented in this study are available on request from the Alzheimer’s Disease Neuroimaging Initiative (ADNI) database: http://adni.loni.usc.edu (accessed on 1 September 2025). Access to the data requires registration and compliance with ADNI’s data use agreement. The authors did not generate any new datasets in this study.

## Abbreviations

The following abbreviations are used in this manuscript:

AD	Alzheimer’s disease
ADNI	Alzheimer’s Disease Neuroimaging Initiative
AI	Artificial intelligence
BBB	blood–brain barrier
CSF	Cerebrospinal fluid
CTRED	CSF erythrocyte burden
CTWHITE	white blood cells of CSF
DBP	Diastolic Blood Pressure
ETIV	estimated intracranial volume
GCP	Google Cloud Platform
MAPres	Mean Arterial Pressure
MBI	Mild Behavioral Impairment
MMSE	Mini Mental Status Examination
MRI	Magnetic Resonance Imaging
NPH	normal pressure hydrocephalus
PCA	Principal component analysis
PET	Positron Emission Tomography
pTau	phosphorylated tau
QSM	Quantitative Susceptibility Mapping
RBC	Red Blood Cells
SBP	Systolic Blood Pressure
SuStain	Subtype and Stage Inference

## References

[1] Scheltens P., De Strooper B., Kivipelto M., Holstege H., Chételat G., Teunissen C.E., Cummings J., van der Flier W.M. (2021). Alzheimer’s Disease. Lancet.

[2] Park J.-Y., Na H.K., Kim S., Kim H., Kim H.J., Seo S.W., Na D.L., Han C.E., Seong J.-K., Alzheimer’s Disease Neuroimaging Initiative (2017). Robust Identification of Alzheimer’s Disease Subtypes Based on Cortical Atrophy Patterns. Sci. Rep..

[3] Poulakis K., Pereira J.B., Muehlboeck J.-S., Wahlund L.-O., Smedby Ö., Volpe G., Masters C.L., Ames D., Niimi Y., Iwatsubo T. (2022). Multi-Cohort and Longitudinal Bayesian Clustering Study of Stage and Subtype in Alzheimer’s Disease. Nat. Commun..

[4] Zhang X., Mormino E.C., Sun N., Sperling R.A., Sabuncu M.R., Yeo B.T.T., Weiner M.W., Aisen P., Weiner M., The Alzheimer’s Disease Neuroimaging Initiative (2016). Bayesian Model Reveals Latent Atrophy Factors with Dissociable Cognitive Trajectories in Alzheimer’s Disease. Proc. Natl. Acad. Sci. USA.

[5] Matsuoka T., Imai A., Narumoto J. (2023). Neuroimaging of Mild Behavioral Impairment: A Systematic Review. PCN Rep..

[6] Angelopoulou E., Androni X., Villa C., Hatzimanolis A., Scarmeas N., Papageorgiou S. (2025). Blood-Based Biomarkers in Mild Behavioral Impairment: An Updated Overview. Front. Neurol..

[7] Matsuoka T., Ueno D., Ismail Z., Rubinstein E., Uchida H., Mimura M., Narumoto J. (2021). Neural Correlates of Mild Behavioral Impairment: A Functional Brain Connectivity Study Using Resting-State Functional Magnetic Resonance Imaging. J. Alzheimer’s Dis..

[8] Rauchmann B.-S., Ersözlü E., Luedecke D., Franzmeier N., Perneczky R. (2025). Multimodal and Longitudinal Characterization of Distinct Tau and Atrophy Clusters in Alzheimer’s Disease Spectrum. Sci. Rep..

[9] Levin F., Ferreira D., Lange C., Dyrba M., Westman E., Buchert R., Teipel S.J., Grothe M.J., For the Alzheimer’s Disease Neuroimaging Initiative (2021). Data-Driven FDG-PET Subtypes of Alzheimer’s Disease-Related Neurodegeneration. Alzheimer’s Res. Ther..

[10] Johnson E.C.B., Bian S., Haque R.U., Carter E.K., Watson C.M., Gordon B.A., Ping L., Duong D.M., Epstein M.P., McDade E. (2023). Cerebrospinal Fluid Proteomics Define the Natural History of Autosomal Dominant Alzheimer’s Disease. Nat. Med..

[11] Tijms B.M., Gobom J., Reus L., Jansen I., Hong S., Dobricic V., Kilpert F., Ten Kate M., Barkhof F., Tsolaki M. (2020). Pathophysiological Subtypes of Alzheimer’s Disease Based on Cerebrospinal Fluid Proteomics. Brain.

[12] Estarellas M., Oxtoby N.P., Schott J.M., Alexander D.C., Young A.L. (2024). Multimodal Subtypes Identified in Alzheimer’s Disease Neuroimaging Initiative Participants by Missing-Data-Enabled Subtype and Stage Inference. Brain Commun..

[13] Hou B., Wen Z., Bao J., Zhang R., Tong B., Yang S., Wen J., Cui Y., Moore J.H., Saykin A.J. (2024). Interpretable deep clustering survival machines for Alzheimer’s disease subtype discovery. Med. Image Anal..

[14] Feng Y., Kim M., Yao X., Lee S., Jeon H., Park H. (2022). Deep Multiview Learning to Identify Imaging-Driven Subtypes in Mild Cognitive Impairment. BMC Bioinform..

[15] Christodoulou R., Vamvouras G., Lorentzen L., Vassiliou E. (2025). Erythrocyte Load in Cerebrospinal Fluid Linked with Hippocampal Atrophy in Alzheimer’s Disease. J. Clin. Med..

[16] (2025). DATAtab Statistics Calculator. https://numiqo.com.

[17] Murray M.E., Graff-Radford N.R., Ross O.A., Petersen R.C., Duara R., Dickson D.W. (2011). Neuropathologically Defined Subtypes of Alzheimer’s Disease with Distinct Clinical Characteristics: A Retrospective Study. Lancet Neurol..

[18] Lunnon K., Mill J. (2013). Epigenetic Studies in Alzheimer’s Disease: Current Findings, Caveats, and Considerations for Future Studies. Am. J. Med. Genet. B Neuropsychiatr. Genet..

[19] Mathys H., Davila-Velderrain J., Peng Z., Gao F., Mohammadi S., Young J.Z., Menon M., He L., Abdurrob F., Jiang X. (2019). Single-Cell Transcriptomic Analysis of Alzheimer’s Disease. Nature.

[20] Wang M., Beckmann N.D., Roussos P., Wang E., Zhou X., Lachmann A., Ma’ayan A., Peters M.A., Neff R., Jia C. (2018). The Mount Sinai Cohort of Large-Scale Genomic, Transcriptomic and Proteomic Data in Alzheimer’s Disease. Sci. Data.

[21] De Souza L.C., Chupin M., Lamari F., Jardel C., Leclercq D., Colliot O., Lehéricy S., Dubois B., Sarazin M. (2012). CSF Tau Markers Are Correlated with Hippocampal Volume in Alzheimer’s Disease. Neurobiol. Aging.

[22] Hsu T.M., Kanoski S.E. (2014). Blood-Brain Barrier Disruption: Mechanistic Links between Western Diet Consumption and Dementia. Front. Aging Neurosci..

[23] .Ashraf A.A., Dani M., So P.-W. (2020). Low Cerebrospinal Fluid Levels of Hemopexin Are Associated With Increased Alzheimer’s Pathology, Hippocampal Hypometabolism, and Cognitive Decline. Front. Mol. Biosci..

[24] Christodoulou R.C., Vamvouras G., Petrou V., Papageorgiou P.S., Pitsillos R., Rivera L., Vassiliou E., Papageorgiou S.G., Solomou E.E., Initiative F.T.A.D.N. (2025). Cerebrospinal Fluid Erythrocyte Burden Amplifies the Impact of PTAU on Entorhinal Degeneration in Alzheimer’s Disease. Biomolecules.

[25] Poulin S.P., Dautoff R., Morris J.C., Barrett L.F., Dickerson B.C. (2011). Amygdala Atrophy Is Prominent in Early Alzheimer’s Disease and Relates to Symptom Severity. Psychiatry Res. Neuroimaging.

[26] Duara R., Barker W. (2022). Heterogeneity in Alzheimer’s Disease Diagnosis and Progression Rates: Implications for Therapeutic Trials. Neurotherapeutics.

[27] Ferreira D., Verhagen C., Hernández-Cabrera J.A., Cavallin L., Guo C.-J., Ekman U., Muehlboeck J.-S., Simmons A., Barroso J., Wahlund L.-O. (2017). Distinct Subtypes of Alzheimer’s Disease Based on Patterns of Brain Atrophy: Longitudinal Trajectories and Clinical Applications. Sci. Rep..

[28] Persson K., Eldholm R.S., Barca M.L., Cavallin L., Ferreira D., Knapskog A.-B., Selbæk G., Brækhus A., Saltvedt I., Westman E. (2017). MRI-Assessed Atrophy Subtypes in Alzheimer’s Disease and the Cognitive Reserve Hypothesis. PLoS ONE.

[29] Van Loenhoud A.C., Van Der Flier W.M., Wink A.M., Dicks E., Groot C., Twisk J., Barkhof F., Scheltens P., Ossenkoppele R., for the Alzheimer’s Disease Neuroimaging Initiative (2019). Cognitive Reserve and Clinical Progression in Alzheimer Disease: A Paradoxical Relationship. Neurology.

[30] Roe C.M., Mintun M.A., D’Angelo G., Xiong C., Grant E.A., Morris J.C. (2008). Alzheimer Disease and Cognitive Reserve: Variation of Education Effect With Carbon 11–Labeled Pittsburgh Compound B Uptake. Arch. Neurol..

[31] Riudavets M.A., Iacono D., Resnick S.M., O’Brien R., Zonderman A.B., Martin L.J., Rudow G., Pletnikova O., Troncoso J.C. (2007). Resistance to Alzheimer’s Pathology Is Associated with Nuclear Hypertrophy in Neurons. Neurobiol. Aging.

[32] Christodoulou R., Christofi G., Pitsillos R., Ibrahim R., Papageorgiou P., Papageorgiou S.G., Vassiliou E., Georgiou M.F. (2025). AI-Based Classification of Mild Cognitive Impairment and Cognitively Normal Patients. J. Clin. Med..

[33] Iaccarino L., Burnham S.C., Dell’Agnello G., Dowsett S.A., Epelbaum S. (2023). Diagnostic Biomarkers of Amyloid and Tau Pathology in Alzheimer’s Disease: An Overview of Tests for Clinical Practice in the United States and Europe. J. Prev. Alzheimer’s Dis..

[34] Gulisano W., Maugeri D., Baltrons M.A., Fà M., Amato A., Palmeri A., D’Adamio L., Grassi C., Devanand D.P., Honig L.S. (2018). Role of Amyloid-β and Tau Proteins in Alzheimer’s Disease: Confuting the Amyloid Cascade. J. Alzheimer’s Dis..

[35] Tahami Monfared A.A., Byrnes M.J., White L.A., Zhang Q. (2022). Alzheimer’s Disease: Epidemiology and Clinical Progression. Neurol. Ther..

[36] Yasar S., Jusue-Torres I., Lu J., Robison J., Patel M.A., Crain B., Carson K.A., Hoffberger J., Batra S., Sankey E. (2017). Alzheimer’s Disease Pathology and Shunt Surgery Outcome in Normal Pressure Hydrocephalus. PLoS ONE.

[37] Müller-Schmitz K., Krasavina-Loka N., Yardimci T., Lipka T., Kolman A.G.J., Robbers S., Menge T., Kujovic M., Seitz R.J. (2020). Normal Pressure Hydrocephalus Associated with Alzheimer’s Disease. Ann. Neurol..

[38] Pyrgelis E.-S., Velonakis G., Papageorgiou S.G., Stefanis L., Kapaki E., Constantinides V.C. (2023). Imaging Markers for Normal Pressure Hydrocephalus: An Overview. Biomedicines.

[39] Kinney J.W., Bemiller S.M., Murtishaw A.S., Leisgang A.M., Salazar A.M., Lamb B.T. (2018). Inflammation as a Central Mechanism in Alzheimer’s Disease. Alzheimer’s Dement. Transl. Res. Clin. Interv..

[40] Christodoulou R.C., Vamvouras G., Sarquis M.D., Petrou V., Papageorgiou P.S., Rivera L., Gonzalez C.M., Rivera G., Papageorgiou S.G., Vassiliou E. (2025). From Microbleeds to Iron: AI Prediction of Cerebrospinal Fluid Erythrocyte Load in Alzheimer’s Disease. J. Clin. Med..

[41] Jeon S., Kang J.M., Seo S., Jeong H.J., Funck T., Lee S.-Y., Park K.H., Lee Y.-B., Yeon B.K., Ido T. (2019). Topographical Heterogeneity of Alzheimer’s Disease Based on MR Imaging, Tau PET, and Amyloid PET. Front. Aging Neurosci..

[42] Vogel J.W., Young A.L., Oxtoby N.P., Smith R., Ossenkoppele R., Strandberg O.T., La Joie R., Aksman L.M., Grothe M.J., The Alzheimer’s Disease Neuroimaging Initiative (2021). Four Distinct Trajectories of Tau Deposition Identified in Alzheimer’s Disease. Nat. Med..

[43] Arnold S.E., Hyman B.T., Betensky R.A., Dodge H.H. (2024). Pathways to Personalized Medicine—Embracing Heterogeneity for Progress in Clinical Therapeutics Research in Alzheimer’s Disease. Alzheimer’s Dement..

[44] Devi G. (2023). A How-to Guide for a Precision Medicine Approach to the Diagnosis and Treatment of Alzheimer’s Disease. Front. Aging Neurosci..

[45] Márquez F., Yassa M.A. (2019). Neuroimaging Biomarkers for Alzheimer’s Disease. Mol. Neurodegener..

[46] Jasodanand V.H., Kowshik S.S., Puducheri S., Romano M.F., Xu L., Au R., Kolachalama V.B. (2025). AI-Driven Fusion of Multimodal Data for Alzheimer’s Disease Biomarker Assessment. Nat. Commun..

[47] Christodoulou R., Woodward A., Pitsillos R., Ibrahim R., Georgiou M. (2025). Artificial Intelligence in Alzheimer’s Disease Diagnosis and Prognosis Using PET-MRI: A Narrative Review of High-Impact Literature Post-Tauvid Approval. J. Clin. Med..

[48] Mandal P.K., Maroon J.C., Samkaria A., Arora Y., Sharma S., Pandey A. (2024). Iron Chelators and Alzheimer’s Disease Clinical Trials. J. Alzheimer’s Dis..

[49] Edwards A.L., Collins J.A., Junge C., Kordasiewicz H., Mignon L., Wu S., Li Y., Lin L., DuBois J., Hutchison R.M. (2023). Exploratory Tau Biomarker Results From a Multiple Ascending-Dose Study of BIIB080 in Alzheimer Disease: A Randomized Clinical Trial. JAMA Neurol..

[50] Ayton S., Barton D., Brew B., Brodtmann A., Clarnette R., Desmond P., Devos D., Ellis K.A., Fazlollahi A., Fradette C. (2025). Deferiprone in Alzheimer Disease: A Randomized Clinical Trial. JAMA Neurol..

[51] Chang Wong E., Chang Chui H. (2022). Vascular Cognitive Impairment and Dementia. Continuum.

[52] Ngandu T., Lehtisalo J., Solomon A., Levälahti E., Ahtiluoto S., Antikainen R., Bäckman L., Hänninen T., Jula A., Laatikainen T. (2015). A 2 Year Multidomain Intervention of Diet, Exercise, Cognitive Training, and Vascular Risk Monitoring versus Control to Prevent Cognitive Decline in at-Risk Elderly People (FINGER): A Randomised Controlled Trial. Lancet.

[53] Martínez-López S., Tabone M., Clemente-Velasco S., González-Soltero M.D.R., Bailén M., De Lucas B., Bressa C., Domínguez-Balmaseda D., Marín-Muñoz J., Antúnez C. (2024). A Systematic Review of Lifestyle-Based Interventions for Managing Alzheimer’s Disease: Insights from Randomized Controlled Trials. J. Alzheimer’s Dis..

[54] Wu C.-K., Fuh J.-L. (2025). A 2025 Update on Treatment Strategies for the Alzheimer’s Disease Spectrum. J. Chin. Med. Assoc..

